# Effectiveness of adjuvant systemic chemotherapy for intermediate-risk stage IB cervical cancer

**DOI:** 10.18632/oncotarget.22437

**Published:** 2017-11-15

**Authors:** Koji Matsuo, Muneaki Shimada, Harushige Yokota, Toyomi Satoh, Hidetaka Katabuchi, Shoji Kodama, Hiroshi Sasaki, Noriomi Matsumura, Mikio Mikami, Toru Sugiyama

**Affiliations:** ^1^ Division of Gynecologic Oncology, Department of Obstetrics and Gynecology, University of Southern California, Los Angeles, CA, USA; ^2^ Norris Comprehensive Cancer Center, University of Southern California, Los Angeles, CA, USA; ^3^ Department of Obstetrics and Gynecology, Tottori University, Tottori, Japan; ^4^ Current affiliation: Department of Obstetrics and Gynecology, Tohoku University, Miyagi, Japan; ^5^ Department of Gynecology, Saitama Cancer Center, Saitama, Japan; ^6^ Department of Obstetrics and Gynecology, University of Tsukuba Faculty of Medicine, Tsukuba, Japan; ^7^ Department of Obstetrics and Gynecology, Faculty of Life Sciences Kumamoto University, Kumamoto, Japan; ^8^ Department of Gynecology, Niigata Cancer Center Hospital, Niigata, Japan; ^9^ Current affiliation: Department of Obstetrics and Gynecology, Niigata Minami Hospital, Niigata, Japan; ^10^ Department of Obstetrics and Gynecology, The Jikei University Kashiwa Hospital, Kashiwa, Japan; ^11^ Current affiliation: Department of Gynecology, Chiba Tokushukai Hospital, Funabashi, Japan; ^12^ Department of Obstetrics and Gynecology, Kyoto University, Kyoto, Japan; ^13^ Current affiliation: Department of Obstetrics and Gynecology, Kindai University Faculty of Medicine, Osaka-Sayama, Japan; ^14^ Department of Obstetrics and Gynecology, Tokai University, Kanagawa, Japan; ^15^ Department of Obstetrics and Gynecology, Iwate Medical University, Iwate, Japan

**Keywords:** cervical cancer, radical hysterectomy, intermediate risk, adjuvant, chemotherapy

## Abstract

**Objective:**

To examine the effectiveness of systemic chemotherapy following radical hysterectomy for women with intermediate-risk stage IB cervical cancer.

**Materials and Methods:**

This is a retrospective analysis of a previously organized nation-wide cohort study examining 6,003 women with stage IB-IIB cervical cancer who underwent radical hysterectomy between 2004 and 2008 in Japan. Survival of 555 women with stage IB cervical cancer in the intermediate-risk group (deep stromal invasion > 50%, large tumor size > 4 cm, and lympho-vascular space invasion [LVSI]) were examined based on adjuvant therapy patterns: chemotherapy alone (*n =* 223, 40.2%), concurrent chemo-radiotherapy (*n =* 172, 31.0%), and radiotherapy alone (*n =* 160, 28.8%).

**Results:**

The most common intermediate-risk pattern was LVSI with deep stromal invasion (*n =* 216, 38.5%). The most common chemotherapeutic choice was taxane/platinum (52.2%). Women with adenocarcinoma/adenosquamous histology were more likely to receive chemotherapy (*P =* 0.03), and intermediate-risk pattern was not associated with chemotherapy use (*P =* 0.11). Women who received systemic chemotherapy had disease-free survival (5-year rate, 88.1% versus 90.2%, adjusted-hazard ratio (HR) 0.98, 95% confidence interval (CI) 0.52–1.83, *P =* 0.94) and cause-specific survival (95.4% versus 94.8%, adjusted-HR 0.85, 95% CI 0.34–2.07, *P =* 0.71) similar to those who received concurrent chemo-radiotherapy on multivariable analysis. Similar results were seen among 329 women with multiple intermediate-risk factors (5-year rates for disease-free survival, chemotherapy versus concurrent chemo-radiotherapy, 87.1% versus 90.2%, *P =* 0.86; and cause-specific survival 94.6% versus 93.4%, *P =* 0.82). Cumulative local-recurrence (*P =* 0.77) and distant-recurrence (*P =* 0.94) risks were similar across the adjuvant therapy types.

**Conclusions:**

Our study suggests that systemic chemotherapy may be an alternative treatment choice for adjuvant therapy in intermediate-risk stage IB cervical cancer.

## INTRODUCTION

Worldwide, cervical cancer was the most common gynecologic malignancy in 2012 [1]. When the gross tumor is confined to the cervix, surgery remains the mainstay of treatment, consisting of radical hysterectomy and pelvic lymphadenectomy [2]. Hysterectomy specimens are useful to identify certain tumor factors associated with an increased risk of disease recurrence. Historically, these surgical-pathological risk factors are grouped into high-risk and intermediate-risk [3–5], and tumor factors in the intermediate-risk group include a large tumor size, deep stromal invasion, and lympho-vascular space invasion (LVSI).

Per the current guidelines set forth by multiple societies, women with early-stage cervical cancer in the intermediate-risk group are recommended to receive postoperative pelvic radiotherapy after surgical treatment in order to reduce the risk of recurrence [6, 7]. While the survival benefit of this adjuvant radiotherapy is supported by level I evidence [4], there is a concern for adverse events related to radiotherapy including genitourinary, hematologic, and gastrointestinal complications [8].

Due to these toxicity profiles of radiotherapy use, systemic chemotherapy has been considered as an alternative treatment option for adjuvant therapy after radical hysterectomy for women in the intermediate-risk group [9–12]. While some studies have suggested the possible utility of systemic chemotherapy, they were conducted with a relatively small sample size or with a lack of an appropriate control arm making their results difficult to interpret.

The objective of this study was to examine survival outcomes of women with intermediate-risk stage IB cervical cancer who received postoperative systemic chemotherapy following radical hysterectomy, and compare these outcomes to those women who received radiation-based therapy.

## RESULTS

**Figure 1:**
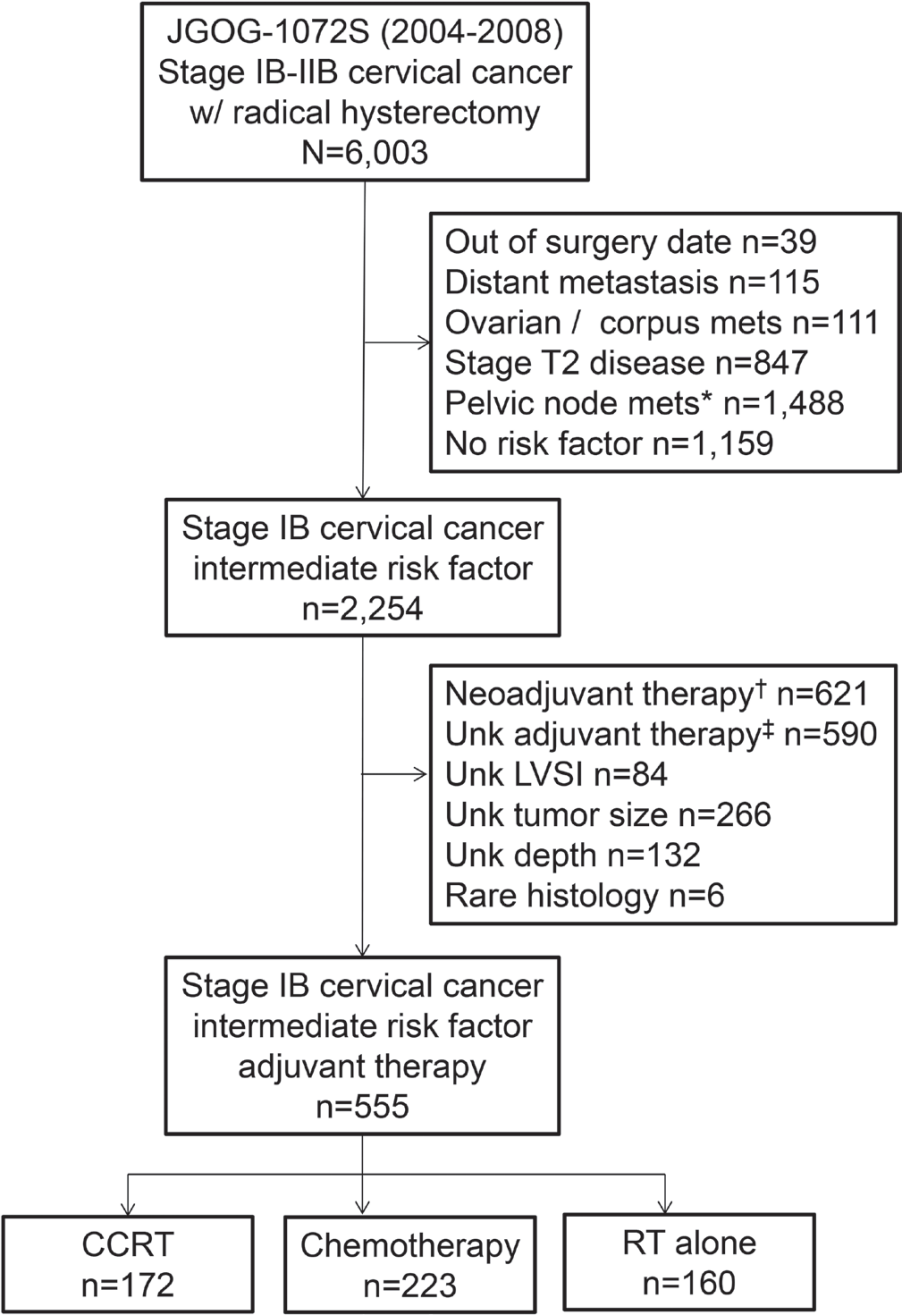
Study selection schema ^*^Including unknown lymph node status. ^†^Including unknown neoadjuvant therapy status. ^‡^Including no adjuvant therapy status. Abbreviations: JGOG, Japanese Gynecologic Oncology Group; unk, unknown; RT, radiotherapy; and CCRT, concurrent chemo-radiotherapy.

The selection schema is shown in Figure 1. Among 6,003 cases in the study cohort, there were 555 cases which met the eligibility criteria for this analysis. Adjuvant therapy patterns included systemic chemotherapy (*n* = 223), concurrent chemo-radiotherapy (CCRT) (*n* = 172), and radiotherapy (RT) alone (*n* = 160).

In the chemotherapy group (Supplementary Table 1), the most common chemotherapy choice was a taxane/platinum-doublet (52.9%) followed by irinotecan-based regimens (19.7%). The median number of administered chemotherapy cycles was 4, and 32.3% received 6 cycles of chemotherapy. The majority of chemotherapy was administered with a 4-week time interval (61.9%). Chemotherapy delay was seen in 37 (16.6%) cases with hematological toxicity being the most common reason for this delay (*n* = 25, 67.6%). Treatment delay was more common among women who received > 3 cycles compared to those who received ≤ 3 cycles (23.7% *versus* 10.6%, *P* = 0.017). There were 23 (10.3%) cases with chemotherapy discontinuation in this study population. In the CCRT group (Supplementary Table 2), the most common radio-sensitizer was cisplatin (*n* = 88, 51.2%) followed by nedaplatin (*n* = 54, 31.4%).

Patient demographics across the three groups are shown in Table 1. Patient age at diagnosis was similar across the groups (*P* = 0.18), and the year of diagnosis was not associated with chemotherapy use (*P* = 0.36). Women with adenocarcinoma / adenosquamous histology were more likely to receive chemotherapy compared to other treatment options: 52.9% for the chemotherapy group, 24.4% for the CCRT group, and 25.6% for the RT group (*P* < 0.001). Women who received chemotherapy had a lower frequency of tumor with deep stromal invasion compared to the other modalities (*P* = 0.02). The frequency of large tumors and LVSI were similar across the three groups (both, *P* > 0.05). Women in the CCRT group had the lowest pelvic lymph node counts among the three groups whereas women in the RT alone had had the lowest para-aortic lymph node counts among the three groups (both, *P* < 0.05).

**Table 1:** Patient demographics (*N* = 555)

Characteristics	CCRT	Chemotherapy	RT alone	*P*-value
No.	*n* ***=*** 172	*n* ***=*** 223	*n* ***=*** 160	
Year				0.36
2004	27 (15.7%)	30 (13.5%)	33 (20.6%)	
2005	26 (15.1%)	44 (19.7%)	28 (17.5%)	
2006	39 (22.7%)	40 (17.9%)	28 (17.5%)	
2007	48 (27.9%)	57 (25.6%)	33 (20.6%)	
2008	32 (18.6%)	52 (23.3%)	38 (23.8%)	
Age (mean ±SD)	46.0 (±10.9)	45.3 (± 11.0)	47.5 (± 12.8)	0.18
Stage				0.32
IB1	130 (75.6%)	181 (81.2%)	130 (81.3%)	
IB2	42 (24.4%)	42 (18.8%)	30 (18.8%)	
Histology				< 0.001
Squamous	130 (75.6%)	105 (47.1%)	119 (74.4%)	
Adenocarcinoma	33 (19.2%)	79 (35.4%)	25 (15.6%)	
Adenosquamous	9 (5.2%)	39 (17.5%)	16 (10.0%)	
Deep stromal invasion				0.02
No	439 (22.7%)	879 (35.4%)	45 (28.1%)	
Yes	133 (77.3%)	144 (64.6%)	115 (71.9%)	
Tumor size				0.13
≤ 4.0 cm	125 (72.7%)	181 (81.2%)	124 (77.5%)	
> 4.0 cm	47 (27.3%)	42 (18.8%)	36 (22.5%)	
LVSI				0.75
Not present	37 (21.5%)	55 (24.7%)	36 (22.5%)	
Present	135 (78.5%)	1768 (75.3%)	124 (77.5%)	
Sampled lymph nodes				
Pelvic (median IQR)	26 (16)	31 (20)	30 (18)	0.001
Para-aortic (median IQR)^*^	7 (8)	8 (9)	5 (7)	0.045
Risk factor patterns				0.09
Deep stroma alone	25 (14.5%)	35 (15.7%)	25 (15.6%)	
Size alone	2 (1.2%)	7 (3.1%)	4 (2.5%)	
LVSI alone	28 (16.3%)	67 (30.0%)	36 (22.5%)	
Deep stroma + size	10 (5.8%)	13 (5.8%)	7 (4.4%)	
LVSI + deep stroma	72 (41.9%)	79 (35.4%)	63 (39.4%)	
LVSI+ size	9 (5.2%)	5 (2.2%)	5 (3.1%)	
All 3 factors	26 (15.1%)	17 (7.6%)	20 (12.5%)	

Mean (± SD), median (interquartile range), or number (%) per column are shown. One-way ANOVA test, chi-square test, or Kruskal-Wallis H test for *P*-values. ^*^Performed in 15 cases in CCRT group, 34 cases in chemotherapy group, and 30 cases for RT group, respectively. Abbreviations: IQR, interquartile range; CCRT, concurrent chemoradiotherapy; and LVSI, lympho-vascular space invasion.

The most common intermediate-risk factor was LVSI (*n* = 427, 76.9%) followed by deep stromal invasion (*n* = 392, 70.6%) and large tumor size (*n* = 125, 22.5%). When cases were stratified by the combination of intermediate-risk factors in the whole cohort, tumors with LVSI and deep stromal invasion was the most common pattern (*n* = 214, 38.6%) followed by LVSI alone (*n* = 131, 23.6%) and deep stromal invasion alone (*n* = 85, 15.3%). There were 63 (11.4%) cases that had all three of these intermediate-risk factors.

**Figure 2:**
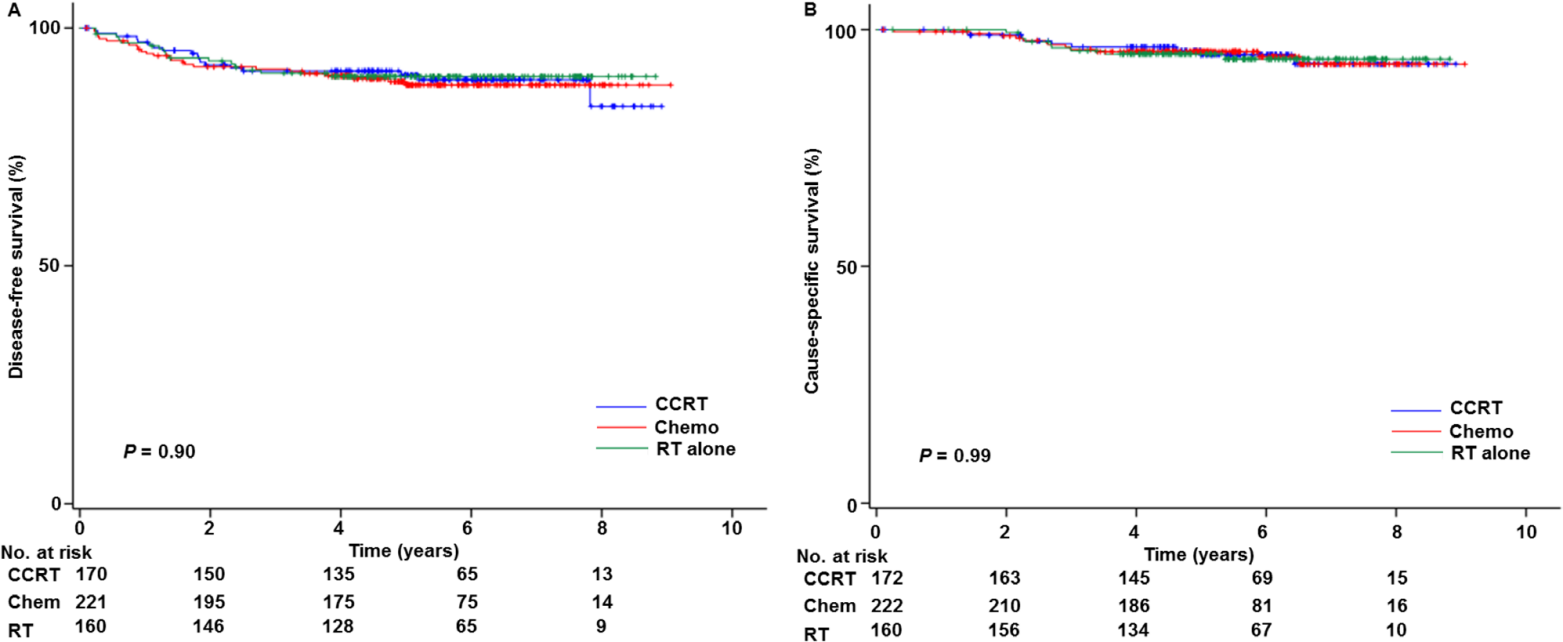
Survival curves based on adjuvant treatment types Log-rank test for adjusted *P*-values. Survival curves based on adjuvant therapy types are shown for: (**A**) disease-free survival and (**B**) cause-specific survival. Abbreviations: CCRT, concurrent chemo-radiotherapy; and RT, whole pelvic radiotherapy alone.

Survival analysis was performed and the median follow-up time of women without survival events was 5.5 years. There were 62 recurrences and 30 deaths due to cervical cancer identified in this study. There were no deaths related to complications from adjuvant chemotherapy or radiotherapy in the cohort. On univariable analysis, adjuvant treatment types were not associated with disease-free survival (5-year rates, 88.0% for chemotherapy group, 90.2% for CCRT group, and 89.8% for RT group, *P* = 0.90; Figure 2A). When the association of adjuvant therapy and disease-free survival was adjusted for other covariates on multivariable models (Table 2), chemotherapy use and CCRT did not differ in disease-free survival (adjusted-hazard ratio (HR) 0.98, 95% confidence interval (CI) 0.52 to 1.84, *P* = 0.95). The systemic chemotherapy group had a similar disease-free survival compared to the RT alone group on adjusted models (data not shown).

**Table 2:** Adjusting models for disease-free survival and adjuvant therapy (*N* = 555)

		Age		Age, histology		Age, histology, risk factors	
Characteristics	No.	HR (95% CI)	*P*-value	HR (95% CI)	*P*-value	HR (95% CI)	*P*-value
Adjuvant type							
CCRT	172	1		1		1	
Chemotherapy alone	223	1.08 (0.59–1.98)	0.81	0.89 (0.48–1.66)	0.71	0.98 (0.52–1.84)	0.95
RT alone	160	0.95 (0.48–1.86)	0.88	0.93 (0.47–1.83)	0.83	0.98 (0.50–1.93)	0.95
Age							
< 50 years	342	1		1		1	
≥ 50 years	213	0.75 (0.43–1.29)	0.329	0.76 (0.44–1.31)	0.32	0.75 (0.44–1.30)	0.31
Histology							
SCC	354			1		1	
Non-SCC	201			1.91 (1.12–3.26)	0.017	2.06 (1.21–3.51)	0.008
Deep stromal invasion							
No	163					1	
Yes	392					1.31 (0.74–2.33)	0.36
Tumor size							
≤4.0 cm	430					1	
>4.0 cm	125					1.20 (0.64–2.25)	0.56
LVSI							
Not present	128					1	
Present	427					3.91 (1.54–9.95)	0.004

An association of adjuvant treatment type and survival outcome was adjusted by survival factors in Cox proportional-hazards regression models. Three models were tested as above. Significant *P*-values were emboldened. Abbreviations: HR, hazard ratio; CI, confidence interval; CCRT, concurrent chemo-radiotherapy; RT, radiotherapy; SCC, squamous cell carcinoma; and LVSI, lympho-vascular space invasion.

Similarly, adjuvant treatment types were not associated with cause-specific survival (5-year rates, 95.3% for chemotherapy group, 94.8% for CCRT group, and 94.8% for RT group, *P* = 0.99; Figure 2B). After adjusting for other covariates for cause-specific survival in multiple models (Table 3), chemotherapy use was not associated with cause-specific survival compared to CCRT (adjusted-HR 0.85, 95% CI 0.34 to 2.08, *P* = 0.71). Likewise, cause-specific survival was similar between the systemic chemotherapy group and the RT alone group on adjusting models (data not shown).

**Table 3:** Adjusting models for cause-specific survival and adjuvant therapy (*N* = 555)

		Age		Age, histology		Age, histology, risk factors	
Characteristics	No.	HR (95% CI)	*P*-value	HR (95% CI)	*P*-value	HR (95% CI)	*P*-value
Adjuvant type							
CCRT	172	1		1		1	
Chemotherapy alone	223	1.03 (0.43–2.44)	0.95	0.85 (0.34–2.08)	0.71	1.02 (0.41–2.54)	0.96
RT alone	160	1.05 (0.42–2.64)	0.92	1.03 (0.41–2.59)	0.95	1.13 (0.44–2.85)	0.80
Age							
< 50 years	342	1		1		1	
≥ 50 years	213	0.77 (0.36–1.65)	0.50	0.79 (0.37–1.68)	0.54	0.81 (0.38–1.73)	0.659
Histology							
SCC	354			1		1	
Non-SCC	201			1.85 (0.87–3.892)	0.11	2.17 (1.02–4.62)	0.045
Deep stromal invasion							
No	163					1	
Yes	392					1.02 (0.47–2.21)	0.97
Tumor size							
≤ 4.0 cm	430					1	
> 4.0 cm	125					2.879 (1.29–6.00)	0.009
LVSI							
Not present	128					1	
Present	427					11.4 (1.52–84.8)	0.018

An association of adjuvant treatment type and survival outcome was adjusted by survival factors in Cox proportional-hazards regression models. Three models were tested as above. Abbreviations: HR, hazard ratio; CI, confidence interval; CCRT, concurrent chemo-radiotherapy; RT, radiotherapy; SCC, squamous cell carcinoma; and LVSI, lympho-vascular space invasion.

When survival was compared between CCRT and RT alone groups, women who received CCRT had disease-free survival (adjusted-HR 1.00, 95% CI 0.53 to 1.90, *P* = 0.99) and cause-specific survival (adjusted-HR 0.82, 95% CI 0.34 to 2.01, *P* = 0.67) similar to those who received RT alone. Among women who received adjuvant chemotherapy, the number of administered cycles was not associated with disease-free survival (5-year rate, 3 *versus* 6 cycles, 89.4% *versus* 89.6%, *P* = 0.84) and cause-specific survival (97.4% *versus* 92.9%, *P* = 0.38). Chemotherapy treatment delay was significantly associated with decreased disease-free survival (70.8% *versus* 91.9%, *P* = 0.001).

Among women who developed recurrent disease (median follow-up time after recurrence 42.6 months for censored cases and 15.3 months for deceased cases), the second remission rates after salvage intervention were statistically similar across the three groups: CCRT group 20%, chemotherapy alone group 30.8%, and 25% for RT alone group (*P* = 0.77).

There were 326 cases in which the tumor exhibited at least two intermediate-risk factors (chemotherapy alone *n* = 114, CCRT *n* = 117, and RT alone *n* = 95). In this sub-group the results were similar to the whole cohort, and adjuvant therapy was not associated with disease-free survival (5-year rates, 86.9% for chemotherapy group, 90.2% for CCRT group, and 85.9% for RT group, *P* = 0.86) and cause-specific survival (5-year rates, 94.5% for chemotherapy group, 93.4% for CCRT group, and 92.2% for RT group, *P* = 0.84). Among 229 cases in which the tumor had only one single intermediate-risk factor, LVSI alone had the lowest disease-free survival compared to other factors although it did not reach statistical significance (5-year rate, 87.8% for LVSI alone, 94.8% for deep stromal invasion alone, and 100% for large tumor alone, *P* = 0.11).

**Figure 3:**
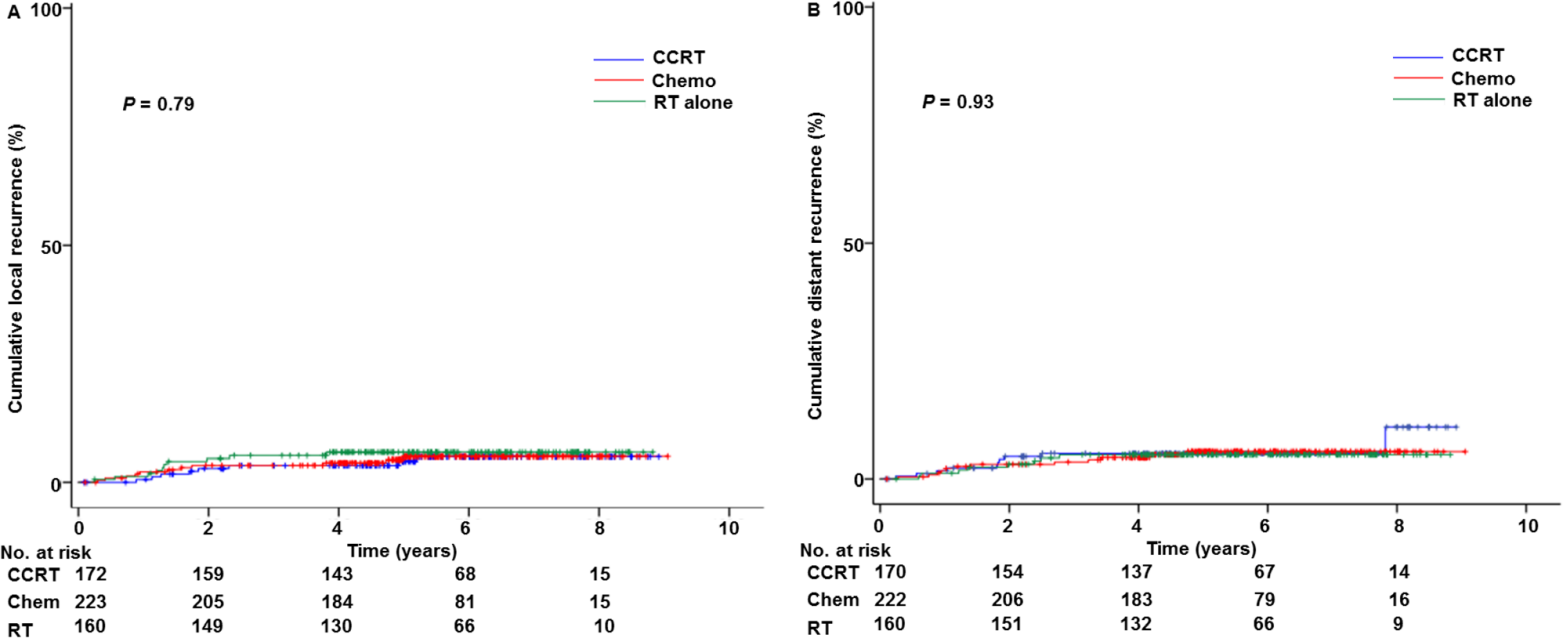
Cumulative incidence curves based on adjuvant treatment types Log-rank test for adjusted *P*-values. Cumulative incidence curves based on adjuvant therapy types are shown for: (**A**) loco-regional recurrence and (**B**) distant recurrence. Abbreviations: CCRT, concurrent chemo-radiotherapy; and RT, whole pelvic radiotherapy alone.

Patterns of recurrence were examined, and local recurrence was seen in 29 cases including 6 cases of vaginal cuff recurrence, while distant recurrence was seen in 33 cases. Adjuvant treatment type was not associated with local recurrence (5-year rates, 5.4% for chemotherapy group, 4.4% for CCRT group, and 6.4% for RT group, *P* = 0.79; Figure 3A) and distant recurrence (5-year rates, 5.9% for chemotherapy group, 5.4% for CCRT group, and 5.1% for RT group, *P* = 0.93; Figure 3B).

Results of propensity score matching are shown in Supplementary Table 3. There were no difference in clinico-pathological factors between the CCRT group and the chemotherapy group (all, *P* > 0.05). Women who received postoperative chemotherapy had disease-free survival (5-year rates, 90.4% versus 89.6%, HR 0.75, 95% CI 0.32 to 1.79, *P* = 0.52) and cause-specific survival (90.4% *versus* 89.6%, HR 0.79, 95% CI 0.21 to 2.92, *P* = 0.72) similar to those who received CCRT.

## DISCUSSION

The key finding of the study is that systemic chemotherapy use was associated with comparable survival compared to radiotherapy-based treatment in women with stage IB cervical cancer and at least one intermediate-risk factor and without a high risk factor. Moreover, our study found that women who had a single intermediate-risk factor with LVSI alone had a survival outcomes similar to those who had multiple intermediate-risk factors.

The effectiveness of systemic chemotherapy as an adjuvant therapy in early-stage cervical cancer in the intermediate-risk group has not been completely studied, and definitive treatment recommendations are currently lacking. Our study findings are similar to prior studies that demonstrated comparable survival outcomes between systemic chemotherapy and CCRT as adjuvant therapy [9, 11]. Because these prior studies were small in sample size (38–47 cases for chemotherapy), our study is more definitive to clarify this association (223 cases for chemotherapy). Therefore, we can respectfully state that postoperative chemotherapy may be as effective as radiation-based therapy.

To date, “classic” criteria of the intermediate-risk group in cervical cancer per the NCCN guidelines require at least two out of the three factors (deep cervical stromal invasion, large tumor, and LVSI) [6]. However, other societies suggest different criteria [13], and the Japan Society of Gynecologic Oncology (JSGO) the intermediate-risk group if any one of the three factors is present in the tumor [7]. We therefore followed the JSGO criteria and included the cases of stage IB cervical cancer with any risk factors as above. However, when we adopted the National Comprehensive Cancer Network (NCCN) guidelines and analyzed a subgroup of cases with multiple risk factors, similar results were demonstrated and chemotherapy use had similar survival outcomes compared to radiotherapy.

Of interest, women whose tumor exhibited only LVSI had survival outcomes similar to those with multiple risk factors (5-year disease-free survival rate for LVSI alone *versus* multiple risk factors, 87.9% *versus* 85.9–90.2%). Recurrence risks were minimal when tumors exhibited only large size or deep cervical stromal invasion (5-year disease-free survival 94.8% and 100%, respectively). Therefore, LVSI alone may need to be considered as an intermediate-risk factor. Further study to validate and prove our results is warranted.

In a sensitivity analysis, we examined survival outcome of women who received CCRT compared to RT alone. The current guidelines for the intermediate-risk group recommend whole pelvic radiotherapy alone without concurrent administration of chemotherapy, and the use of CCRT is optional [6, 7]. In review of the literature, there were multiple cohort studies examined the effectiveness of CCRT over RT alone for the intermediate-risk group [14, 15], suggesting improved survival with CCRT use. Conversely, other investigators concluded that CCRT does not improve survival when compared to radiotherapy alone in the intermediate-risk group [16]. Our study also did not demonstrate a difference in survival between CCRT and RT alone groups. Currently, there is an ongoing phase III randomized controlled trial comparing CCRT *versus* RT alone in women with surgically treated intermediate-risk early-stage cervical cancer (GOG-263) [17]. The estimated time for the completion of accrual is December 2020, and this trial will ultimately answer the utility of CCRT for this patient population.

The benefit of offering systemic chemotherapy over radiotherapy is to minimize complications from pelvic irradiation administered after radical pelvic surgery. This rationale was based on a general consensus that demonstrated high complication rates with pelvic irradiation after radical hysterectomy compared to surgery alone [18]. In the intermediate-risk group, one prior retrospective study reported a higher adverse event rate in the CCRT group compared to the chemotherapy group [11]. Our study did not have information to assess postoperative complications. To date, there is no head-to-head trial directly comparing efficacy and adverse events between adjuvant chemotherapy and radiotherapy in intermediate-risk early-stage cervical cancer. In Japan, JGOG is going to launch a phase III trial comparing adjuvant chemotherapy and CCRT in women with high-risk stage IB-IIB cervical cancer (AFTER trial). Although the patient population is not an intermediate-risk group, this trial will answer the question regarding the adverse event related to adjuvant therapy.

Strengths of the study included a sample size that is one of the largest in the literature. In addition, the dataset is considerably clear with our rigorous inclusion and exclusion criteria, making interpretation of the results more reliable. However, there are multiple limitations in this study. First, this is a retrospective study that may miss confounding factors in the analysis. For example, we do not know the treatment allocation for chemotherapy *versus* other treatment types. Indeed, there are various practice patterns for adjuvant therapy in intermediate-risk early-stage cervical cancer in Japan [19], and this can be a major drawback of the study resulting in selection bias. Second, there are multiple chemotherapy regimens and administered cycles used in the chemotherapy group (heterogeneity), and we were not able to recommend any specific chemotherapy choice over others.

Weaknesses of the study included that our study population had a fairly favorable survival outcome with the limited number of survival events despite the adequate median follow-up of more than five years. For this reason, an adjustment model with five covariates for cause-specific survival may result in over-adjustment given the number of cervical cancer mortality events. However, two other adjustment models for cause-specific survival were consistent to support the current findings, and this association of chemotherapy and cause-specific survival is likely true. While central pathology review was not performed to confirm that intermediate-risk criteria was met, these surgical-pathological factors are well-established histopathological findings and thus inter-observer-variability is less likely to differ across the pathologists.

The intermediate-risk criteria for deep stromal invasion was recorded as outer versus inner half and tumor size was recorded for > 4 versus ≤ 4 cm in this surgical database per the JSGO criteria, and thus, we were not able to apply the Gynecologic Oncology Group (GOG) or Korean criteria for the intermediate-risk in cases where the depth of invasion was defined as outer third [4, 20]. In this study, we used the JSGO criteria because this study was conducted in the society designated institutions and the validation of the society criteria has not been tested in large-scale study population. However, when we examined the cases with any two or more risk factors, survival outcome of our study population seems comparable to that seen in the GOG-92 trial (2-year disease-free survival rate 88%) [4].

In summary, our study suggests that systemic chemotherapy can be an alternative effective treatment choice as adjuvant therapy for women with intermediate-risk stage IB cervical cancer. This option can be particularly suitable in patients who are not the best candidates for radiotherapy such as those with pelvic adhesive disease, intraoperative abdomino-pelvic injury, radiation intolerance, and non-compliance for the radiation treatment schedule. Moreover, because increased chemotherapy cycles did not improve survival, and women in the intermediate-risk group have a generally good prognosis, the utility of a reduction in the number of chemotherapy cycles may be considered to minimize the toxicity of chemotherapy. Finally, the current intermediate-risk criteria per the JSGO guidelines need to be revised, as the present criteria may result in overtreatment in cases where the prognosis is generally favorable. Based on our results, at least two risk factors as well as LVSI alone may meet criteria for stratification into the intermediate-risk category. Establishing stricter criteria weighing treatment benefits and risks are warranted.

## MATERIALS AND METHODS

### Eligibility

This was a retrospective analysis of the previously organized nation-wide large-scale observational study conducted in 116 Japanese Gynecologic Oncology Group (JGOG) designated institutions [20–22]. We collected consecutive cases of women with stage IB-IIB cervical cancer who underwent a radical hysterectomy between January 1, 2004 and December 31, 2008. The study period for the data acquisition was between October 1, 2012 and February 28, 2013. Institutional Review Board approval was obtained at Tottori University, which served as the host institution, and JGOG-participating institutions reviewed the protocol and obtained Institutional Review Board approval as indicated.

Eligibility criteria for this study were women with stage IB cervical cancer who met criteria for intermediate-risk disease, and received adjuvant therapy following type III radical hysterectomy and pelvic lymphadenectomy. Per the JSGO treatment guidelines for cervical cancer, the intermediate-risk group is defined as cervical cancer in which the tumor is confined to the cervix without parametrial or lymph node involvement, and exhibits any one of the following three factors: large cervical tumor > 4 cm, deep cervical stromal invasion (outer half), and LVSI [7]. We limited the histology types only to squamous cell carcinoma, adenocarcinoma, and adenosquamous for comparison with a prior study [4]. Women were excluded from the study if high-risk criteria were present (pelvic lymph node metastasis, parametrial tumor involvement, and surgical margin tumor involvement) or if no surgical-pathological risk factor was identified. We also excluded women with tumor involving in para-aortic lymph nodes, ovaries, or uterine corpus. Women who received neoadjuvant therapy, unknown adjuvant therapy, or received a battery of both systemic chemotherapy and radiotherapy were also excluded.

### Clinical information

Clinical and tumor information abstracted from medical and pathological records included age, histologic subtype, clinical and pathological stages, tumor size, pelvic and para-aortic lymph node status (including the number sampled), parametrial involvement, deep stromal invasion, LVSI, uterine corpus involvement, ovarian involvement, and the presence of distant metastasis. Adjuvant treatment information included the following three modalities: concurrent chemo-radiotherapy with pelvic irradiation and weekly chemotherapy (CCRT group), systemic chemotherapy alone (chemotherapy group), and whole pelvic radiotherapy alone (RT group). Among women who received chemotherapy, the type of chemotherapy, the number of administered cycles, and toxicity were recorded.

Survival information included disease-free and cause-specific survival. Disease-free survival was defined as the time interval between the hysterectomy and the first recurrence. Cause-specific survival was defined as the time interval between the hysterectomy date and death due to cervical cancer. The patients were censored if patients were alive at the last follow-up or had died due to another cause. Among women who developed recurrent disease, locations of recurrence were grouped into local recurrence (vaginal cuff and/or pelvis) and distant recurrence (any site other than local).

### Statistical analysis

The primary interest of this analysis was to examine survival of women with intermediate-risk stage IB cervical cancer who received systemic chemotherapy compared to those who received CCRT. The secondary interest of analysis was to assess recurrence patterns based on adjuvant therapy types. A sensitivity analysis was performed to examine cases with at least two intermediate-risk factors by adopting the NCCN guidelines [6]. In addition, another sensitivity analysis was performed to compare survival of the CCRT group to the RT alone group per an ongoing phase III clinical trial examining intermediate-risk cervical cancer (GOG-263) [17]. Similarly, a sensitivity analysis was performed to examine survival of the systemic chemotherapy group compared to the RT alone group as radiotherapy alone is the current standard adjuvant therapy for patients in the intermediate-risk group [4].

The statistical significance of continuous variables among multiple groups was assessed by either the one-way ANOVA test or by the Kruskal-Wallis H test as appropriate. Statistical significance of categorical and ordinal variables was assessed by the chi-square test. The Kaplan-Meier method was used to construct survival curves and cumulative risk curves [23], and statistical significance between these curves was determined by the log-rank test. Cox proportional hazard regression models were used to assess the independent association of adjuvant therapy and survival by adjusting for *a priori* survival factors [24]. In this study, we tested three different models. The first model was adjusted for age alone (< 50 *versus* ≥ 50 years), the second model was adjusted for age and histology type (squamous *versus* non-squamous), and the third model was adjusted for age, histology, and histo-pathological factors (tumor size, depth of myometrial tumor invasion, and LVSI). The magnitude of statistical significance was expressed with an adjusted-HR and 95% CI.

We also performed a propensity score matching to adjust the background differences between the chemotherapy group and the CCRT group. Propensity score for chemotherapy use was computed for each case determined by multivariable logistic regression analysis. In the model, year of diagnosis, age, stage, histology types, extent of lymphadenectomy and tumor factors (deep stromal invasion, tumor size, and LVSI). An automated algorithm was used for one-to-one matching between the two groups (cutoff, 1%).

All statistical analyses were based on two-side hypothesis, and a *P*-value of less than 0.05 was considered significant. Statistical Package for Social Sciences (IBM SPSS, version 24.0, Armonk, NY, USA) was used for the analysis. The STROBE guidelines for a retrospective observational study were consulted to outline this study [25].

## SUPPLEMENTARY MATERIALS TABLES


