# Supplementary material for: Effectiveness of adjuvant systemic chemotherapy for intermediate-risk stage IB cervical cancer
Source: Oncotarget. 2017 Nov 15;8(63):106866–75. doi: 10.18632/oncotarget.22437 (PMC5739780; doi:10.18632/oncotarget.22437)
Supplement: Supplementary file 1 [file oncotarget-08-106866-s001.pdf]

# Effectiveness of adjuvant systemic chemotherapy for intermediate-risk stage IB cervical cancer

## SUPPLEMENTARY MATERIALS

**Supplementary Table 1: Treatment types in the Chemotherapy group (*n* = 223)**

| Characteristic | Number | Percent |
|---|---|---|
| **Chemotherapy regimen** | | |
| Carboplatin + paclitaxel | 70 | 31.4 |
| Cisplatin + paclitaxel | 33 | 14.8 |
| Nedaplatin + irinotecan | 25 | 11.2 |
| Cisplatin + irinotecan | 14 | 6.3 |
| Carboplatin + docetaxel | 13 | 5.8 |
| Cisplatin + etoposide + mitomycin C | 9 | 4.0 |
| Cisplatin + bleomycin + vincristine + mitomycin C | 8 | 3.6 |
| Fluorouracil | 7 | 3.1 |
| Nedaplatin + ifosfamide | 5 | 2.2 |
| Nedaplatin alone | 4 | 1.8 |
| Carboplatin + paclitaxel + pirarubicin | 3 | 1.3 |
| Cisplatin + fluorouracil | 3 | 1.3 |
| Irinotecan + mitomycin C | 3 | 1.3 |
| Cisplatin + doxorubicin + cyclophosphamide | 3 | 1.3 |
| Nedaplatin + ifosfamide + peplomycin | 3 | 1.3 |
| Cisplatin + mitomycin C + peplomycin + vincristine | 3 | 1.3 |
| Nedaplatin + ifosfamide + bleomycin | 2 | 0.9 |
| Cisplatin + ifosfamide + epirubicin | 2 | 0.9 |
| Other regimens | 13 | 5.8 |
| **Chemotherapy cycle** | | |
| 1–2 | 15 | 6.7 |
| 3–4 | 105 | 47.1 |
| 5–6 | 91 | 40.8 |
| > 6 | 2 | 0.9 |
| n/a | 10 | 4.5 |
| **Chemotherapy interval** | | |
| 3 weeks | 73 | 32.7 |
| 4 weeks | 138 | 61.9 |
| n/a | 12 | 5.4 |
| **Chemotherapy delay** | | |
| No | 170 | 76.2 |
| Yes | 37* | 16.6 |
| n/a | 16 | 7.2 |
| **Chemotherapy discontinuation** | | |
| No | 171 | 76.7 |
| Yes | 23 | 10.3 |
| n/a | 29 | 13.0 |

**Supplementary Table 2: Treatment types in the CCRT group (*n* = 172)**

| Characteristic | Number | Percent |
|---|---|---|
| **Radiosensitizer type** | | |
| Cisplatin | 88 | 51.2 |
| Nedaplatin | 54 | 31.4 |
| Fluorouracil | 5 | 2.9 |
| Cisplatin + fluorouracil | 3 | 1.7 |
| Others | 6 | 3.5 |
| n/a | 16 | 9.3 |
| **Radiation dose (Gy)** | | |
| 50.0 | 59 | 34.3 |
| 50.4 | 42 | 24.4 |
| 45.0 | 30 | 17.4 |
| 46.0 | 22 | 12.8 |
| 48.6 | 4 | 2.3 |
| 51.0 | 4 | 2.3 |
| 44.0 | 1 | 0.6 |
| 45.8 | 1 | 0.6 |
| 46.8 | 1 | 0.6 |
| 50.6 | 1 | 0.6 |
| n/a | 7 | 4.1 |

**Supplementary Table 3: Patient demographics after propensity score matching**

| Characteristics | CCRT | Chemotherapy | *P*-value |
|---|---|---|---|
| No. | *n* = 99 | *n* = 99 | |
| Year | | | 0.97 |
| 2004 | 14 (14.1%) | 14 (14.1%) | |
| 2005 | 15 (15.2%) | 18 (18.2%) | |
| 2006 | 23 (23.2%) | 20 (20.2%) | |
| 2007 | 27 (27.3%) | 28 (28.3%) | |
| 2008 | 20 (20.2%) | 19 (19.2%) | |
| Age (mean ±SD) | 45.0 (±10.4) | 45.9 (±10.7) | 0.54 |
| Stage | | | 0.99 |
| IB1 | 77 (77.8%) | 78 (78.8%) | |
| IB2 | 22 (22.2%) | 21 (21.2%) | |
| Histology | | | 0.89 |
| Squamous | 66 (66.7%) | 63 (63.6%) | |
| Adenocarcinoma | 25 (25.3%) | 28 (28.3%) | |
| Adenosquamous | 8 (8.1%) | 8 (8.1%) | |
| Deep stromal invasion | | | 0.99 |
| No | 27 (27.3%) | 27 (27.3%) | |
| Yes | 72 (72.7%) | 72 (72.7%) | |
| Tumor size | | | 0.61 |
| ≤ 4.0 cm | 75 (75.8%) | 79 (79.8%) | |
| > 4.0 cm | 24 (24.2%) | 20 (20.2%) | |
| LVSI | | | 0.60 |
| Not present | 23 (23.2%) | 19 (19.2%) | |
| Present | 76 (76.8%) | 80 (80.8%) | |
| Sampled lymph nodes | | | |
| Pelvic (median IQR) | 29.5 (18) | 28 (17) | 0.93 |

Mean (± SD), median (interquartile range), or number (%) per column are shown. Student *t* test, Fisher exact test, chi-square test, or Mann-Whitney *U* test for *P*-values. Abbreviations: CCRT, concurrent chemoradiotherapy; and LVSI, lympho-vascular space invasion.
